# Enamel Defects Associated With Dentin Sialophosphoprotein Mutation in Mice

**DOI:** 10.3389/fphys.2021.724098

**Published:** 2021-09-24

**Authors:** Tian Liang, Qian Xu, Hua Zhang, Suzhen Wang, Thomas G. H. Diekwisch, Chunlin Qin, Yongbo Lu

**Affiliations:** ^1^Department of Biomedical Sciences and Center for Craniofacial Research and Diagnosis, Texas A&M University College of Dentistry, Dallas, TX, United States; ^2^Department of Periodontics and Center for Craniofacial Research and Diagnosis, Texas A&M University College of Dentistry, Dallas, TX, United States

**Keywords:** tooth development, enamel, amelogenesis, ameloblasts, cell differentiation, mineralization, dentin sialophosphoprotein

## Abstract

Dentin sialophosphoprotein (DSPP) is an extracellular matrix protein that is highly expressed in odontoblasts, but only transiently expressed in presecretory ameloblasts during tooth development. We previously generated a knockin mouse model expressing a mouse equivalent (DSPP, p.P19L) of human mutant DSPP (p.P17L; referred to as “*Dspp^P19L/+^*”), and reported that *Dspp^P19L/+^* and *Dspp^P19L/P19L^* mice manifested a dentin phenotype resembling human dentinogenesis imperfecta (DGI). In this study, we analyzed pathogenic effects of mutant P19L-DSPP on enamel development in *Dspp^P19L/+^* and *Dspp^P19L/P19L^* mice. Micro-Computed Tomography (μCT) analyses of 7-week-old mouse mandibular incisors showed that *Dspp^P19L/P19L^* mice had significantly decreased enamel volume and/or enamel density at different stages of amelogenesis examined. Acid-etched scanning electron microscopy (SEM) analyses of mouse incisors demonstrated that, at the mid-late maturation stage of amelogenesis, the enamel of wild-type mice already had apparent decussating pattern of enamel rods, whereas only minute particulates were found in *Dspp^P19L/+^* mice, and no discernible structures in *Dspp^P19L/P19L^* mouse enamel. However, by the time that incisor enamel was about to erupt into oral cavity, distinct decussating enamel rods were evident in *Dspp^P19L/+^* mice, but only poorly-defined enamel rods were revealed in *Dspp^P19L/P19L^* mice. Moreover, μCT analyses of the mandibular first molars showed that *Dspp^P19L/+^* and *Dspp^P19L/P19L^* mice had a significant reduction in enamel volume and enamel density at the ages of 2, 3, and 24weeks after birth. Backscattered and acid-etched SEM analyses revealed that while 3-week-old *Dspp^P19L/+^* mice had similar pattern of enamel rods in the mandibular first molars as age-matched wild-type mice, no distinct enamel rods were observed in *Dspp^P19L/P19L^* mice. Yet neither *Dspp^P19L/+^* nor *Dspp^P19L/P19L^* mice showed well-defined enamel rods in the mandibular first molars by the age of 24weeks, as judged by backscattered and acid-etched SEM. *In situ* hybridization showed that *DSPP* mRNA level was markedly reduced in the presecretory ameloblasts, but immunohistochemistry revealed that DSP/DSPP immunostaining signals were much stronger within the presecretory ameloblasts in *Dspp* mutant mice than in wild-type mice. These results suggest that mutant P19L-DSPP protein caused developmental enamel defects in mice, which may be associated with intracellular retention of mutant DSPP in the presecretory ameloblasts.

## Introduction

Tooth development involves a series of sequential interactions that occur between dental epithelium and underlying mesenchyme (Thesleff, 2003; Balic and Thesleff, 2015). The dental epithelium differentiates into ameloblasts, which form enamel. Enamel formation occurs in three major stages, including presecretory, secretory, and maturation stages (Warshawsky and Smith, 1974; Hu et al., 2007; Moradian-Oldak, 2012; Balic and Thesleff, 2015). During the presecretory stage, the dental epithelium differentiates into presecretory ameloblasts. As the presecretory ameloblasts differentiate into secretory ameloblasts, they acquire a special apical structure known as “Tomes’ process.” The secretory ameloblasts with Tomes’ processes produce and secrete a specific set of proteins to form organic enamel matrix, which is then partially replaced by minerals during the secretory stage. The full thickness of enamel is achieved at the end of the secretory stage, and the organic matrix is completely removed and replaced by minerals during the maturation stage. The underlying mesenchyme differentiates into odontoblasts, which form dentin located directly underneath enamel in the crown of a tooth (Balic and Thesleff, 2015).

Mutations in the genes encoding enamel matrix proteins as well as other proteins involved in enamel formation result in a group of inherited enamel defects, known as “Amelogenesis Imperfecta (AI; Smith et al., 2017).” Based on the phenotypic defects of enamel, AI may be classified into hypoplastic and hypomineralized forms (Hu et al., 2007; Smith et al., 2017). Hypoplastic AI is characterized by the formation of a thin but mineralized layer of enamel that results from an insufficient apposition of enamel during the secretory stage of amelogenesis. In contrast, hypomineralized AI is distinguished by the formation of a full thickness but soft layer of enamel caused by a failure in enamel maturation. Hypomineralized AI can be further subclassified into hypomaturation and hypocalcified forms (Hu et al., 2007; Smith et al., 2017). Hypomaturation AI is due to an incomplete removal of the organic enamel matrix proteins that separate adjacent enamel crystals, whereas hypocalcified AI is caused by an insufficient deposition of minerals, particularly calcium ions.

Dentin sialophosphoprotein (DSPP) is a non-collagenous extracellular matrix protein and is a member of the SIBLING (Small integrin-binding ligand N-linked glycoprotein) family (Fisher et al., 2001; Fisher and Fedarko, 2003). It is highly expressed in odontoblasts, but transiently expressed in differentiating ameloblasts, as its transcripts are only observed in presecretory ameloblasts and early secretory ameloblasts during tooth development (D'Souza et al., 1997; Ritchie et al., 1997; Begue-Kirn et al., 1998; MacDougall et al., 1998; Bleicher et al., 1999). DSPP is synthesized as a single large protein, which is subsequently processed into an amino-terminal fragment called dentin sialoprotein (DSP) and a carboxyl-terminal fragment known as dentin phosphoprotein (DPP; MacDougall et al., 1997; Sun et al., 2010; von Marschall and Fisher, 2010; Zhu et al., 2012). DSP is a proteoglycan containing two glycosaminoglycan chains (Ritchie et al., 1994; Zhu et al., 2010; Yamakoshi et al., 2011), whereas DPP is a highly phosphorylated and very acidic protein (Butler et al., 1983; George et al., 1996; Ritchie and Wang, 1996). In addition, a third cleaved fragment, named “dentin glycoprotein (DGP),” is identified in porcine and is derived from the middle region of DSPP (Yamakoshi et al., 2005).

Mutations in the *DSPP* gene in humans affect tooth development, resulting in an inheritable autosomal dominant dental disorder. Consistent with its high level of expression in odontoblasts, the primary dental defect associated with DSPP mutations in humans is the formation of various types of abnormal dentin (Kim and Simmer, 2007). Based on the phenotypic differences, the dental defects caused by DSPP mutations may be classified into dentinogenesis imperfecta (DGI) Type II (OMIM #125490), characterized by pulp chamber obliteration, DGI Type III (OMIM #125500), featured by pulp chamber enlargement and thinner dentin, and dentin dysplasia (DD) Type II (OMIM #125420), which manifests relatively mild dental defects (Shields et al., 1973; MacDougall et al., 2006; Kim and Simmer, 2007). Similarly, *Dspp* ablation in mice causes a defective dentin mineralization, resulting in a tooth phenotype similar to that observed in human DGI type III patients (Sreenath et al., 2003). In addition to dentin, enamel formation may be affected by DSPP mutations in DGI patients (Lee et al., 2011; Wang et al., 2011; Bloch-Zupan et al., 2016; Taleb et al., 2018). However, very limited studies have been done to understand how mutant DSPP proteins interfere with amelogenesis to date.

We previously generated a mouse model (referred to as “*Dspp^P19L/+^*” mice), that expressed a mutant DSPP, in which the proline residue at position 19 was replaced by a leucine residue (p.P19L; Liang et al., 2019). The proline residue at position 19 in mouse DSPP is the second amino acid residue from the signal peptide cleavage site, and it corresponds to the proline residue P17 in human DSPP. Our findings demonstrated that *Dspp^P19L/+^* and *Dspp^P19L/P19L^* mice manifested a DGI Type III-like phenotype at younger age, and acquired a DGI Type II-like defect as they grew older, which is similar to the dental phenotype of human patients carrying the corresponding p.P17L mutation (Li et al., 2012; Lee et al., 2013; Porntaveetus et al., 2019). In this study, we reported the ultrastructural changes in enamel as well as the molecular changes in differentiating ameloblasts in *Dspp^P19L/+^* and *Dspp^P19L/P19L^* mice. We showed that the *Dspp* mutant mice had reduced enamel formation, delayed enamel maturation, and ultrastructural enamel defects; and we demonstrated that such enamel defects may be associated with an accumulation of mutant DSPP protein within the presecretory ameloblasts.

## Materials and Methods

### Generation of *Dspp^P19L/+^* and *Dspp^P19L/P19L^* Mice

Generation and genotyping of *Dspp^P19L/+^* and *Dspp^P19L/P19L^* mice was described in our previous report (Liang et al., 2019). All mice were maintained on a C57BL/6 genetic background on a 12h light/dark cycle with free access to water and standard pelleted food. Both male and female mice were used for analyses of the enamel phenotypes, as there was no phenotypic difference between sexes for each genotype. All animal procedures were approved by the Institutional Animal Care and Use Committee of Texas A&M University College of Dentistry (Dallas, TX, United States).

### Plain X-Ray Radiography and Micro-Computed Tomography

**Figure 1:**
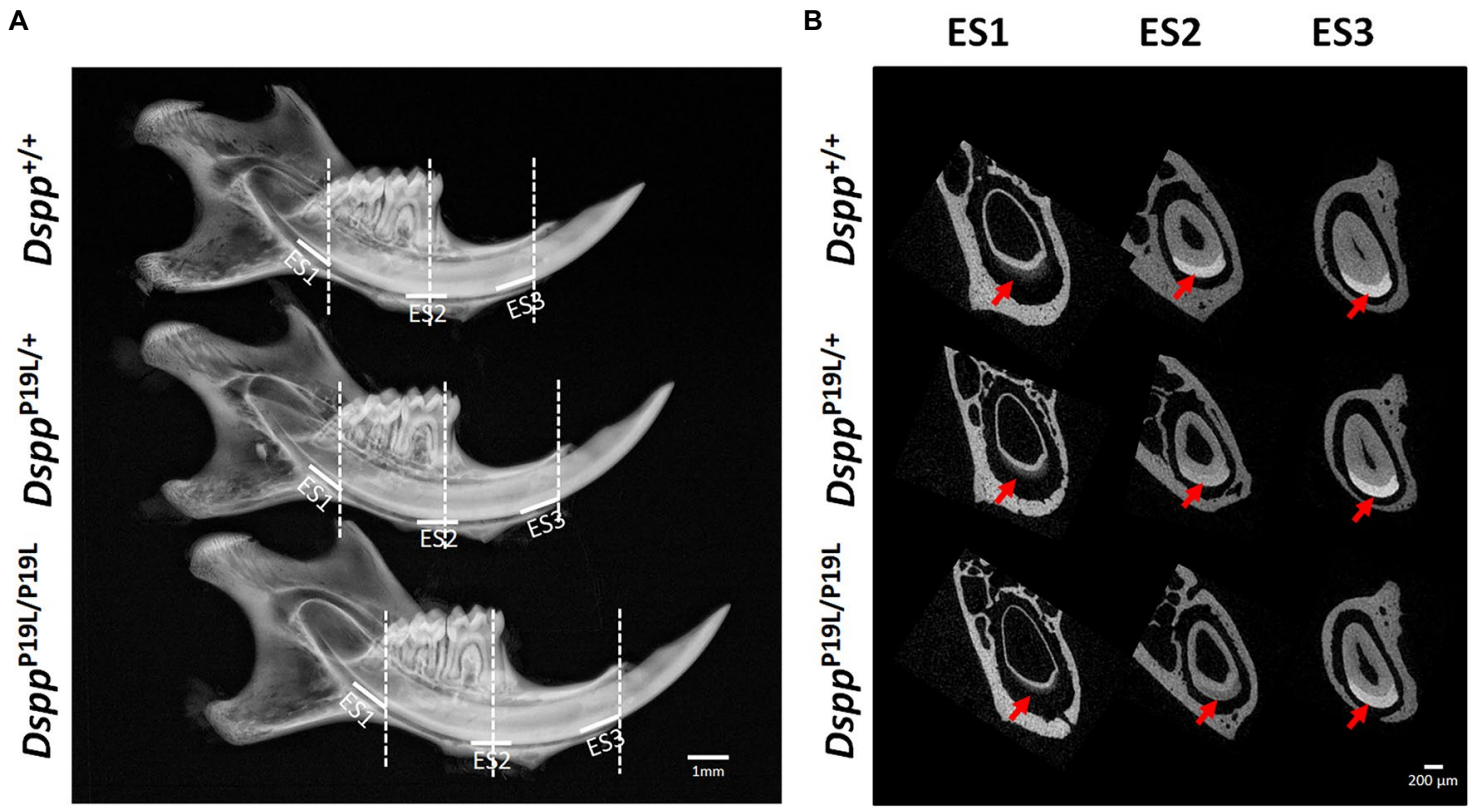
Gross overview of the tooth phenotype of *Dspp^P19L/+^* and *Dspp^P19L/P19L^* mice. **(A)** Representative plain x-ray radiographic images of the mandibles of 7-week-old wild-type *Dspp^+/+^*, heterozygous *Dspp^P19L/+^* and homozygous *Dspp^P19L/P19L^* mice. The white horizontal lines denote the enamel segments, ES1, ES2, and ES3, that were quantified by μCT analyses. ES1 corresponds to the late secretory stage amelogenesis; ES2 corresponds to mid-late maturation stage amelogenesis; and ES3 is the enamel segment that has undergone extensive maturation and is about to erupt. The dashed vertical white lines in **(A)** mark the locations of reconstructed trans-axial μCT images shown in **(B)**. Red arrows indicate the mandibular incisor enamel in the trans-axial images. Scale bars: 1mm in A; 200μm in B.

The left mandibles from 2-, 3-, 7-, and 24-week-old *Dspp^+/+^*, *Dspp^P19L/+^*, and *Dspp^P19L/P19L^* mice were dissected and processed for plain x-ray radiography and/or μCT analyses, as previously described (Bouxsein et al., 2010; Liang et al., 2019). Seven-week-old mouse mandibles were first evaluated using a plain X-ray radiography system (Faxitron MX-20DC 12; Tucson, AZ, United States) to obtain an overall morphological assessment of the mandibular incisors; they were then scanned with a Scanco μCT35 imaging system (Scanco Medical, Brüttisellen, Switzerland) at three regions that contain the mandibular incisor enamel segment 1 (ES1), ES2, and ES3, respectively (Figure 1A). ES1 is a 1-mm-long incisor enamel segment located immediately distal to the distal aspect of the third molar crown, representing the late secretory stage amelogenesis; ES2 is a 1-mm-long incisor enamel segment located right underneath the proximal root of first molar, representing the mid-late maturation stage amelogenesis; ES3 is a 1-mm-long incisor enamel segment that is located below the labial alveolar crest, that is about to erupt into oral cavity, and that has undergone extensive maturation (enamel mineral density reaches its peak). For the morphometric analyses of ES1, ES2, and ES3, the enamel layer was manually delineated to separate enamel from the subjacent dentin; and a threshold of 120 was then chosen to exclude any soft tissues/ameloblasts. The left mandibles from 2-, 3-, and 24-week-old *Dspp^+/+^*, *Dspp^P19L/+^*, and *Dspp^P19L/P19L^* mice were scanned for mandibular first molars. For three-dimensional (3D) construction and morphometric analyses of mandibular first molars, the whole teeth were outlined, and an appropriate threshold was determined for each age (a threshold of 515 for 2-week-old mice, 563 for 3-week-old mice, and 600 for 24-week-old mice), based on the visual comparisons to include enamel but not adjacent tissues. All morphometric parameters were evaluated using the μCT built-in software (Bouxsein et al., 2010). Data obtained from 3 to 5 independent mice for each group were used for quantitative analysis.

### Resin-Casted Backscattered and Acid-Etched Scanning Electron Microscopy

The 3-, 7-, and 24-week-old left mandible samples were processed for resin-casted backscattered and acid-etched SEM analysis, as described previously (Gibson et al., 2013; Zhang et al., 2018; Liang et al., 2019). Briefly, the mandible samples were dehydrated in gradient ethanol (from 70 to 100%) and xylene before embedded in methyl methacrylate (MMA). For resin-casted backscattered SEM analysis of the molars, the buccal-lingual sections crossing the middle of the proximal root of mandibular first molars were chosen. The cut surface was polished and dehydrated, followed by gold coating. Scanning was performed in backscattered electronic shadow (BES) mode in a JEOL JSM-6010 LA SEM (JEOL, Japan). Following resin-casted backscattered SEM analysis, the molar sections were further processed for acid-etched SEM analysis. The coating particles were removed, and sample surface was re-polished and etched with 10% phosphoric acid for 7–14s, followed by immersing in 5% sodium hypochlorite for 20min twice. The samples were gold-coated and scanned in secondary electron image (SEI) mode in the same SEM. For acid-etched SEM analysis of 7-week-old mandibular incisors, the mandibles were cross sectioned to obtain sections at the levels of ES2 and ES3, which were subsequently processed for acid-etched SEM analysis as the molar sections. Two independent mice were analyzed for molars or incisors for each genotype of 3-, 7-, and 24-week-old mice.

### Sample Processing and Histological Analysis

The right mandibles from 1-week-old *Dspp^+/+^*, *Dspp^P19L/+^*, and *Dspp^P19L/P19L^* mice were harvested and fixed in freshly prepared 4% paraformaldehyde in diethyl pyrocarbonate (DEPC)-treated 0.1M phosphate-buffered saline (pH 7.4) at 4°C overnight and then decalcified in 15% ethylenediaminetetraacetate (EDTA) solution (pH 7.4) at 4°C for 3days. The decalcified samples were dehydrated, and embedded in paraffin following standard histological procedures. The tissue blocks were cut into serial sagittal sections at a thickness of 5μm, which were used for Hematoxylin and Eosin (H&E) staining, and other histological analyses, as previously described (Zhang et al., 2018; Liang et al., 2019).

### *In situ* Hybridization

*In situ* hybridization (ISH) was performed to detect DSPP transcripts, as previously described (Meng et al., 2015; Liang et al., 2019). Briefly, RNA probes for mouse DSPP were labeled with digoxigenin (DIG) by using an RNA Labeling Kit (Roche, Indianapolis, IN, United States), according to the manufacturer’s instruction. The DIG-labeled RNA probes were used to hybridize with the DSPP transcripts in the mandibular first molars. After hybridization, the hybridized DIG-labeled RNA probes were detected by an enzyme-linked immunoassay with an alkaline phosphatase-conjugated anti-DIG antibody (1:2000; Roche), and an NBT/BCIP (nitro blue tetrazolium/5-bromo-4-chloro-3-indolyl-phosphate) substrate (Roche, Mannheim, Germany). The sections were counterstained with nuclear fast red (Sigma, Saint Louis, MO, United States). At least three individual samples were analyzed.

### Immunohistochemistry

Immunohistochemistry (IHC) was carried out to assess DSP/DSPP, amelogenin (AMEL), and ameloblastin (AMBN) proteins, as previously described (Meng et al., 2015; Li et al., 2019; Liang et al., 2019). Briefly, sections were incubated with a rabbit anti-DSP polyclonal antibody (recognizing both DSP and full-length DSPP), a mouse monoclonal antibody raised against the full-length human AMEL (Santa Cruz Biotechnology; 1:500), or a rabbit polyclonal antibody generated against the C-terminus of mouse AMBN (Santa Cruz Biotechnology; 1:800), followed by incubating in a biotinylated anti-rabbit IgG or anti-mouse IgG secondary antibody (1:200, Vector Laboratories, Burlingame, CA, United States). The immunostaining signals were visualized using the DAB (3,3′-diaminobenzidine) kit (Vector Laboratories), according to the manufacturer’s instructions. The sections were counterstained with methyl green (Sigma). At least three individual samples were analyzed. The same concentrations of nonimmune rabbit IgG or mouse IgG were used to replace the polyclonal or monoclonal antibodies as negative controls.

### Statistical Analysis

One-way ANOVA was conducted to compare the differences among three groups. If significant differences were found by One-way ANOVA, Bonferroni method was used as *post hoc*. The quantified results were represented as mean±SD. *p*<0.05 was considered statistically significant.

## Results

### Delayed Incisor Enamel Maturation in *Dspp^P19L/+^* and *Dspp^P19L/P19L^* Mice

**Figure 2:**
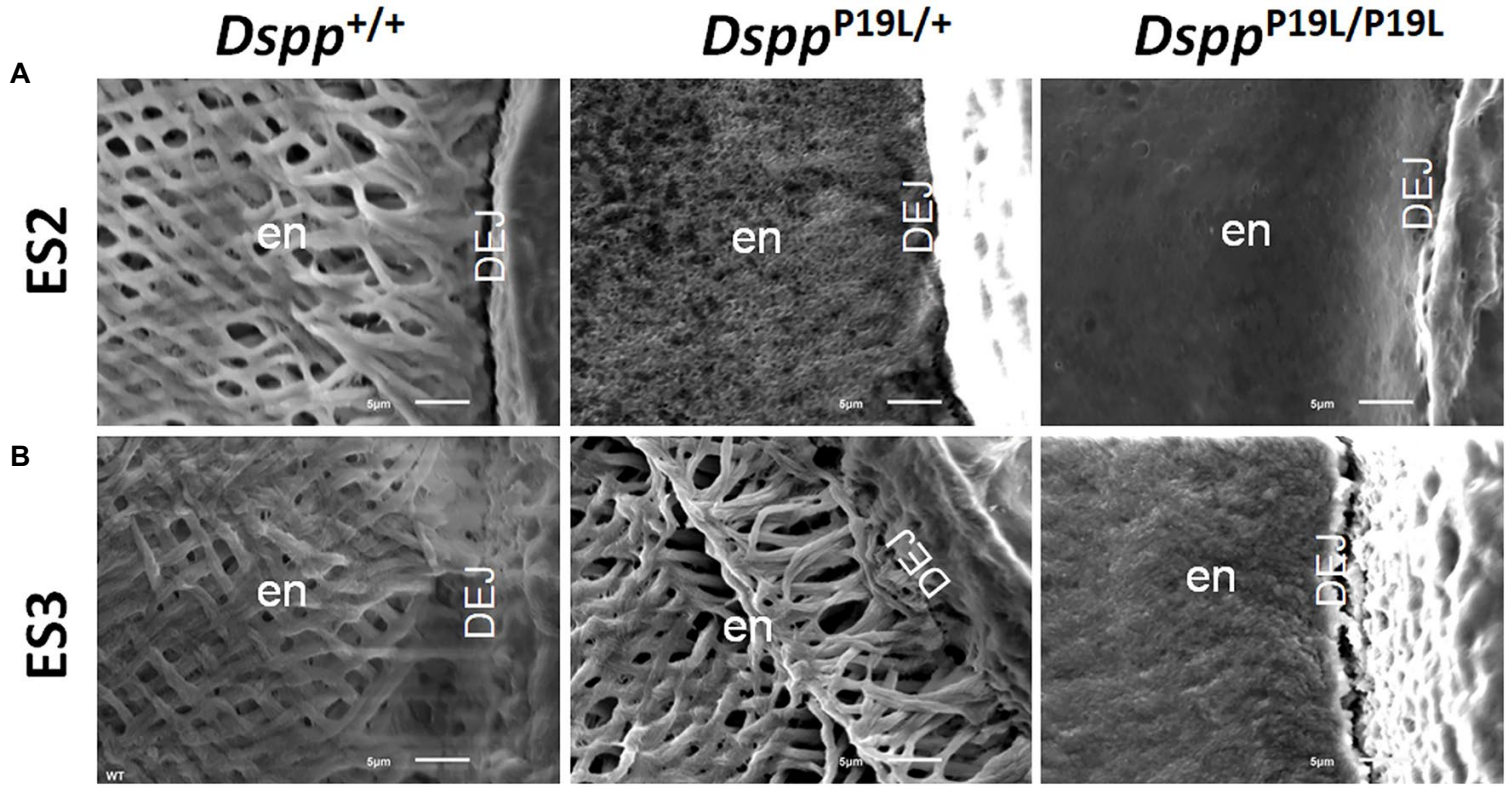
Acid-etched scanning electron microscopy (SEM) analyses of the mandibular incisors. Shown are the acid-etched SEM images of the mandibular incisors of 7-week-old wild-type *Dspp^+/+^*, heterozygous *Dspp^P19L/+^* and homozygous *Dspp^P19L/P19L^* mice. All images are the cross sections obtained at the levels of ES2 **(A)** and ES3 **(B)**. Note that the enamel from *Dspp^+/+^* mice showed decussating pattern of enamel rods at both ES2 and ES3 levels. In *Dspp^P19L/+^* mouse incisors, even though only minute particulates were observed at the level of ES2, distinct decussating enamel rods were present at the level of ES3. In the *Dspp^P19L/P19L^* mouse incisors, there was a complete absence of any discernible structures at the level of ES2, and only poorly-defined enamel rods were found at the level of ES3. en, enamel and DEJ, dentinoenamel junction. Scale bars: 5μm.

Rodent incisors continuously grow and erupt, so that different stages of amelogenesis can be evaluated in the same incisor (Robinson et al., 1977; Smith and Nanci, 1989; Smith et al., 2011; Schmitz et al., 2014). We first examined the mandibular incisor phenotype of 7-week-old *Dspp^P19L/+^* and *Dspp^P19L/P19L^* mice. Plain x-ray radiography demonstrated that *Dspp^P19L/+^* and *Dspp^P19L/P19L^* mice had no apparent difference in the overall appearance of the mandibular incisors, compared to the age-matched *Dspp^+/+^* control mice (Figure 1A). The trans-axial μCT images demonstrated that *Dspp^P19L/+^* and *Dspp^P19L/P19L^* mice had reduced radiopacity in enamel, compared to *Dspp^+/+^* mice at the late secretory stage (ES1) and at the mid-late maturation stage (ES2; Figure 1B). Such reduced radiopacity was more prominent at the mid-late maturation stage and was also more severe in *Dspp^P19L/P19L^* mice (Figure 1B). However, for the enamel segment (ES3), which was immediately before eruption, *Dspp^P19L/+^* and *Dspp^P19L/P19L^* mice achieved a radiopacity that was very close to that of *Dspp^+/+^* mice (Figure 1B). Quantitative μCT analyses showed that *Dspp^P19L/P19L^* mice had a significant reduction in enamel volume at all three stages of amelogenesis examined, whereas *Dspp^P19L/+^* mice only displayed reduced enamel volume at the level of ES2, compared to *Dspp^+/+^* control mice (Table 1). In addition, both *Dspp^P19L/+^* and *Dspp^P19L/P19L^* mice had significantly reduced enamel density at the level of ES2, compared to *Dspp^+/+^* mice (Table 1). We also analyzed the ultrastructural changes in the mandibular incisor enamel. Acid-etched scanning electron microscopy (SEM) analyses demonstrated that, at the mid-late maturation stage of amelogenesis, the wild-type mice already had apparent decussating pattern of enamel rods, whereas only minute particulates were found in *Dspp^P19L/+^* mice, and no discernible structures in *Dspp^P19L/P19L^* mice (Figure 2A). However, by the stage that the incisor enamel is about to erupt into the oral cavity, distinct decussating enamel rods were evident in *Dspp^P19L/+^* mice, but only poorly-defined enamel rods were revealed in *Dspp^P19L/P19L^* mice (Figure 2B). Taken together, these results demonstrated that *Dspp^P19L/+^* and *Dspp^P19L/P19L^* mice showed reduced incisor enamel formation, and delayed enamel maturation.

**Table 1:** Micro-Computed Tomography analysis of 7-week-old mouse mandibular incisor enamel.

		ES1	ES2	ES3
Enamel volume (mm^3^)	*Dspp^+/+^*	0.0656±0.0150	0.0821±0.0029	0.0844±0.0034
	*Dspp^P19L/+^*	0.0473±0.0052	0.0748±0.0045^a^	0.0807±0.0060
	*Dspp^P19L/P19L^*	0.0297±0.0073^a^^,^^b^	0.0647±0.0073^a^	0.0737±0.0015^a^
Enamel density (mg/cm^3^ HA)	*Dspp^+/+^*	309.84±41.09	1621.64±39.04	1792.34±27.20
	*Dspp^P19L/+^*	300.53±33.98	1308.87±192.25^a^	1731.77±67.96
	*Dspp^P19L/P19L^*	251.09±32.32	1006.10±388.08^a^	1558.77±189.55

*n=3–4; ES, enamel segment; values are mean±SD*.

a *Statistically different from Dspp^+/+^ (p<0.05)*.

b *Statistically different from Dspp^P19L/+^ (p<0.05)*.

### Reduced Molar Enamel Formation and Accelerated Molar Enamel Attrition in *Dspp^P19L/+^* and *Dspp^P19L/P19L^* Mice

**Figure 3:**
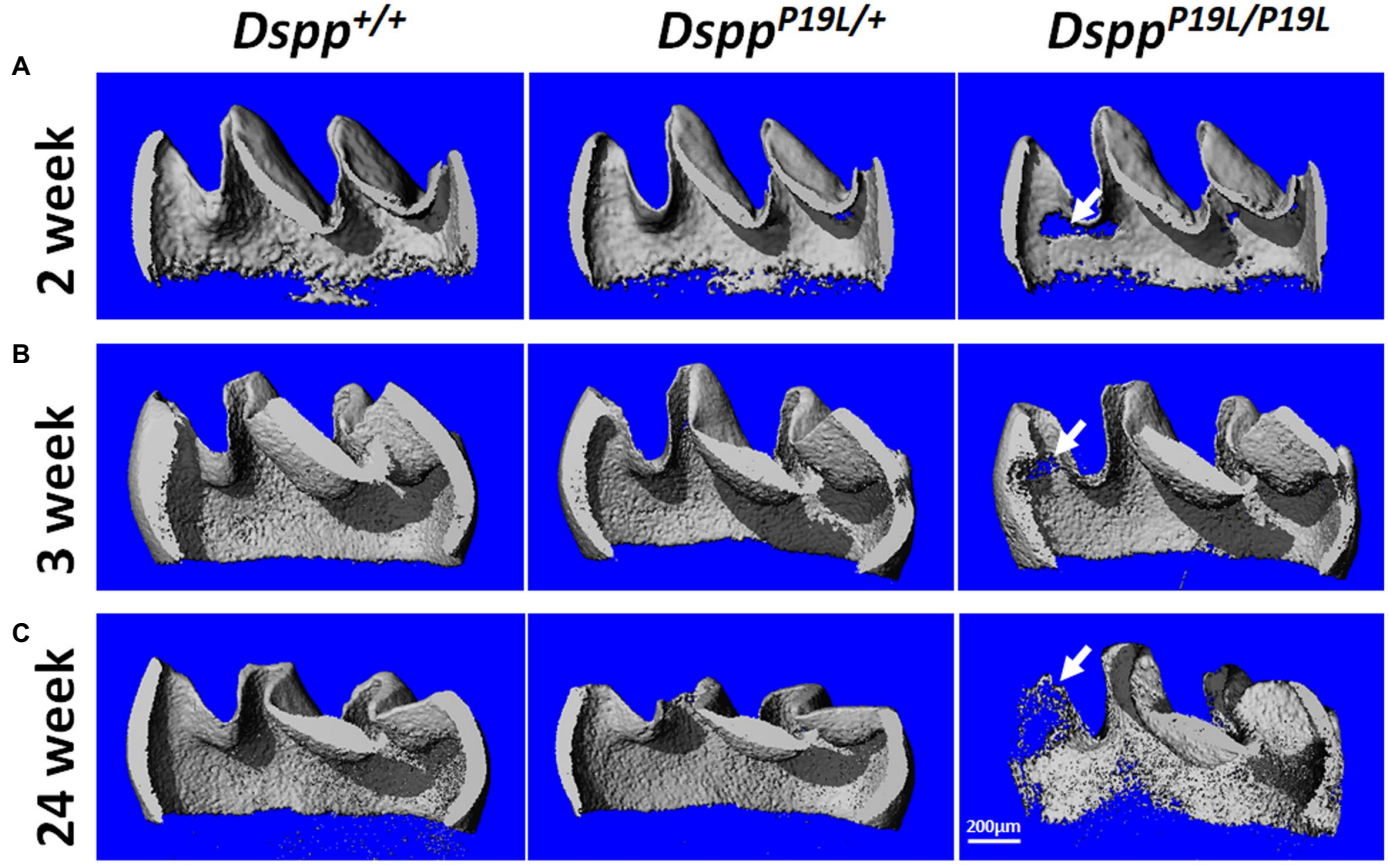
Micro-Computed Tomography (μCT) analyses of the mandibular first molars. Shown are the representative 3D reconstructed μCT images (sagittal sections) of enamel of the mandibular first molars of 2-week-old **(A)**, 3-week-old **(B)**, and 24-week-old **(C)** wild-type *Dspp^+/+^*, heterozygous *Dspp^P19L/+^*, and homozygous *Dspp^P19L/P19L^* mice. The enamel was poorly formed in *Dspp^P19L/+^* and *Dspp^P19L/P19L^* mice, particularly in *Dspp^P19L/P19L^* mice (arrows), at the age of 2weeks. There was also a severe loss of enamel in the *Dspp^P19L/P19L^* mice (arrows) with age, in comparison to age-matched *Dspp^+/+^* and *Dspp^P19L/^* mice. Scale bar: 200μm.

Next, we analyzed the mandibular first molars (M1) of *Dspp^P19L/+^* and *Dspp^P19L/P19L^* mice at postnatal 2weeks (M1 at the beginning of eruption phase before it is exposed to oral cavity; Komiyama et al., 2013), 3weeks (M1 at the beginning of post-eruptive phase when it reaches the occlusal plane; Komiyama et al., 2013), and 24weeks (when M1 has undergone substantial attrition). In contrast to incisors, rodent molars have limited growth as no more enamel will be formed once enamel formation is complete. Reconstructed 3D μCT images showed that the enamel was poorly formed in *Dspp^P19L/+^* and *Dspp^P19L/P19L^* mice, particularly in *Dspp^P19L/P19L^* mice, at the age of 2weeks before the teeth were exposed to oral cavity (Figure 3A). Moreover, *Dspp^P19L/P19L^* mice showed a more severe loss of enamel with age after tooth eruption, compared with age-matched *Dspp^+/+^* and *Dspp^P19L/+^* mice (Figures 3B,C). Quantitative μCT analyses confirmed that *Dspp^P19L/+^* and *Dspp^P19L/P19L^* mice had a significant reduction in both enamel volume and enamel density of the mandibular first molars at the age of 2weeks, compared to *Dspp^+/+^* mice (Table 2). The enamel volumes and densities of *Dspp^P19L/+^* and *Dspp^P19L/P19L^* mouse molars remained significantly lower than those of age-matched *Dspp^+/+^* mice when the teeth reached occlusion at the age of 3weeks (Table 2). Even though all three groups of mice showed reduced molar enamel volumes with age due to attrition, *Dspp^P19L/+^* and *Dspp^P19L/P19L^* mouse molars apparently showed a more severe loss of enamel volume (with a loss of 15.74 and 33.48% total enamel volume, respectively) from the age of 3 to 24weeks, compared to the *Dspp^+/+^* mice (with a loss of 7.48% total enamel volume; Table 2). In contrast to enamel volume, the molar enamel densities increased with age among all three groups of mice; however, the enamel densities of *Dspp^P19L/+^* and *Dspp^P19L/P19L^* mouse molars remained significantly lower, compared to that of *Dspp^+/+^* mice at the age 24weeks (Table 2). These data suggest that the molar enamel was less formed and hypomineralized in *Dspp^P19L/+^* and *Dspp^P19L/P19L^* mice and was subject to rapid attrition with age after teeth reach occlusion.

**Table 2:** Micro-Computed Tomography analysis of mandibular first molar enamel.

		2Weeks	3Weeks	24Weeks
Enamel volume (mm^3^)	*Dspp^+/+^*	0.1643±0.0068	0.1617±0.0130	0.1496±0.0092
	*Dspp^P19L/+^*	0.1384±0.0109^a^	0.1245±0.0225^a^	0.1049±0.0140^a^
	*Dspp^P19L/P19L^*	0.0987±0.0007^a^^,^^b^	0.1129±0.0147^a^	0.0751±0.0187^a^
Enamel density (mg/cm^3^ HA)	*Dspp^+/+^*	1709.6854±18.2788	1779.91±31.70	1899.49±34.74
	*Dspp^P19L/+^*	1644.3523±25.0770^a^	1723.43±39.34^a^	1833.88±17.12^a^
	*Dspp^P19L/P19L^*	1582.3119±30.2098^a^^,^^b^	1729.06±30.36^a^	1819.08±12.19^a^

*n=3–5; values are mean±SD*.

a *Statistically different from Dspp^+/+^ (p<0.05)*.

b *Statistically different from Dspp^P19L/+^ (p<0.05)*.

### Ultrastructural Changes in Molar Enamel in *Dspp^P19L/+^* and *Dspp^P19L/P19L^* Mice

**Figure 4:**
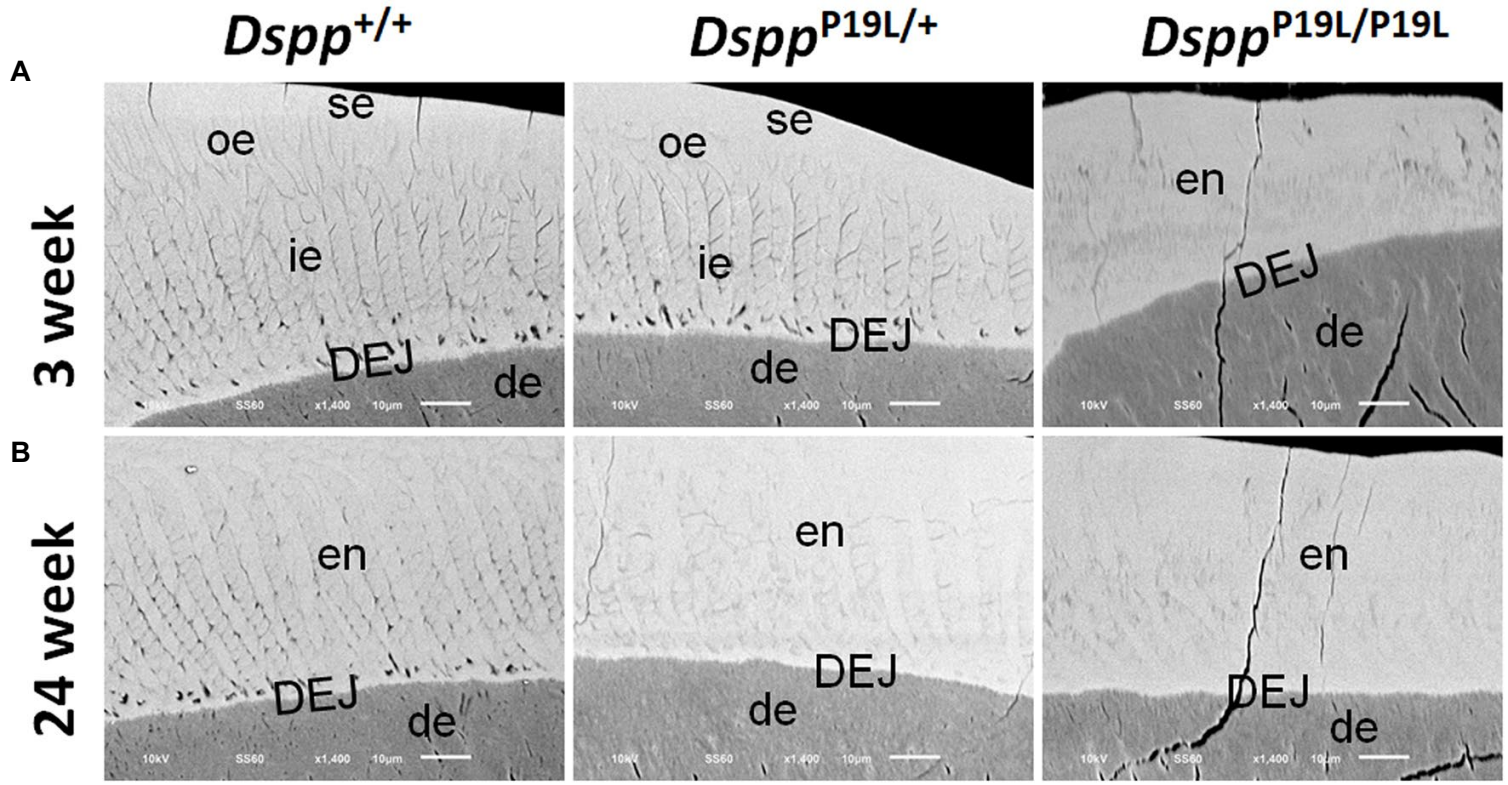
Backscattered SEM analyses of the mandibular first molars. Shown are the backscattered SEM images of the mandibular first molars of 3- **(A)** and 24- **(B)** week-old wild-type *Dspp^+/+^*, heterozygous *Dspp^P19L/+^* and homozygous *Dspp^P19L/P19L^* mice. All images are the longitudinal sections and buccal sides of the mandibular first molars. Three-week-old *Dspp^+/+^* mice showed a decussating pattern of enamel rods in the inner enamel (ie), a parallel pattern in the outer enamel (ore), and were rod-free in the superficial enamel (se). Three-week-old *Dspp^P19L/+^* mice showed a similar arrangement of enamel rods as the age-matched wild-type mice, but there were no discernable enamel rods in 3-week-old *Dspp^P19L/P19L^* mice. By 24weeks, the decussating enamel rods remain in *Dspp^+/+^* mice. However, the enamel rod boundaries became blurred in *Dspp^P19L/+^* mice, whereas only poorly-defined enamel rods were observed in *Dspp^P19L/P19L^* mice. ie, inner enamel; oe, outer enamel; en, enamel; se, superficial enamel; de, dentin; and DEJ, dentinoenamel junction. Scale bars: 10μm.

**Figure 5:**
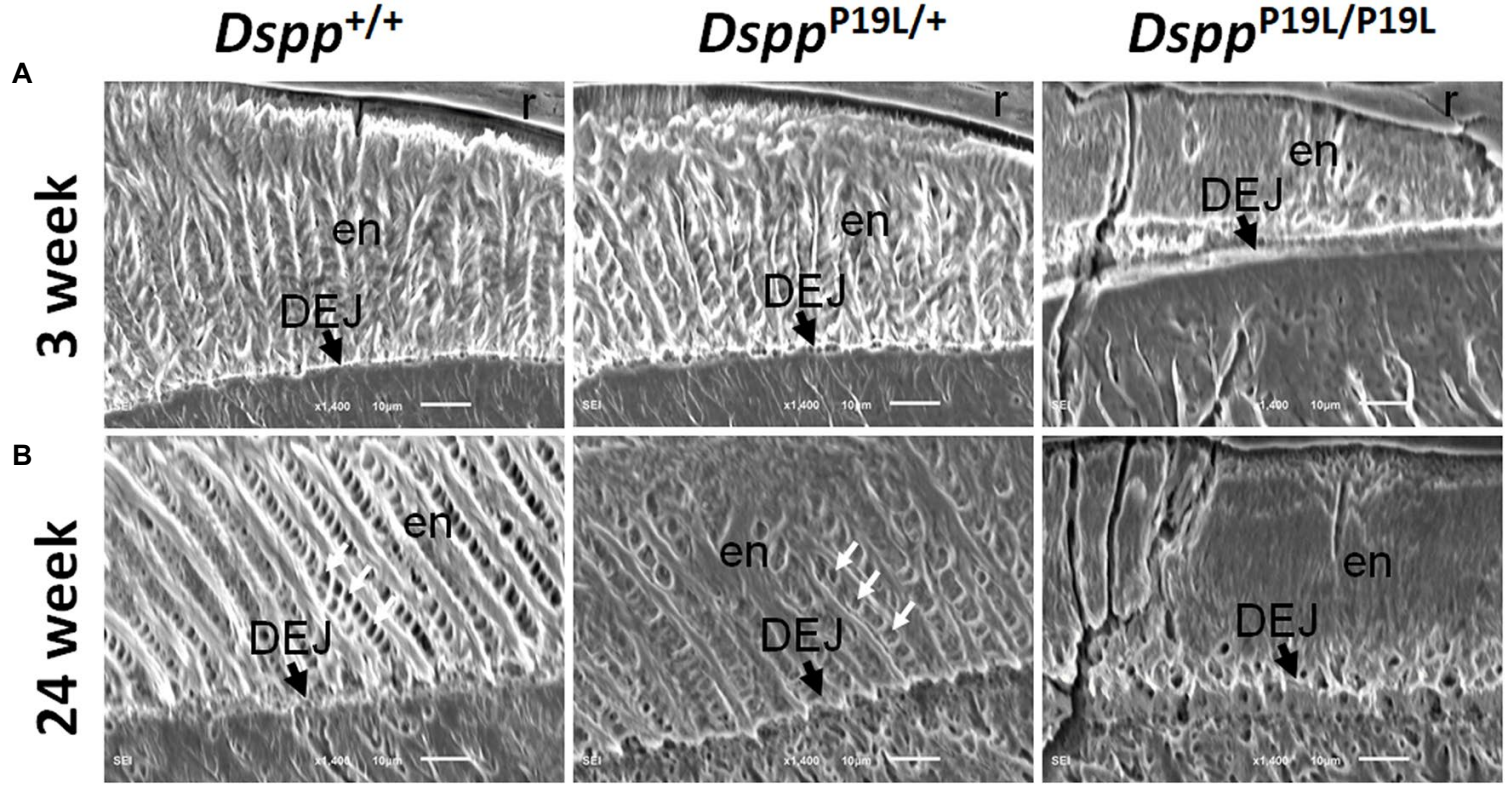
Acid-etched SEM analyses of the mandibular first molars. Shown are the acid-etched SEM images of the mandibular first molars of 3- **(A)** and 24- **(B)** week-old wild-type *Dspp^+/+^*, heterozygous *Dspp^P19L/+^* and homozygous *Dspp^P19L/P19L^* mice. All images are the longitudinal sections and buccal sides of the mandibular first molars. At the age of 3weeks, *Dspp^P19L/+^* mice showed a similar acid-etching pattern in enamel (en) as the wild-type mice. At the age of 24weeks, *Dspp^+/+^* mice presented an acid-etching pattern, with the cross-sectioned enamel rods (pointed by white arrows) preferentially removed by acid; although *Dspp^P19L/+^* mice displayed a similar pattern, the cross-sectioned enamel rods (pointed by white arrows) were not as well-defined as the wild-type mice. There were no recognizable enamel rods in *Dspp^P19L/P19L^* mice at both ages. The dentinoenamel junction (DEJ) appeared to be different in *Dspp^P19L/P19L^* mice, compared to age-matched *Dspp^+/+^* and *Dspp^P19L/+^* mice. en, enamel; DEJ, dentinoenamel junction; and r, resin. Scale bars: 10μm.

We then examined the ultrastructural changes in the molar enamel by backscattered and acid-etched SEM analyses, as previously described (Liang et al., 2019). Backscattered SEM analysis demonstrated that 3-week-old *Dspp^+/+^* mice showed a decussating pattern of enamel rods in the inner enamel, a parallel pattern in the outer enamel and were rod-free in the superficial enamel (Figure 4A). Three-week-old *Dspp^P19L/+^* mice showed a similar organization of enamel rods as age-matched *Dspp^+/+^* mice, but there was a complete lack of clearly defined enamel rods in 3-week-old *Dspp^P19L/P19L^* mice (Figure 4A). By 24weeks, the decussating pattern enamel rods remained in *Dspp^+/+^* mice (Figure 4B). However, the enamel rod boundaries became blurred in *Dspp^P19L/+^* mice, whereas only poorly-defined enamel rods were observed in *Dspp^P19L/P19L^* mice (Figure 4B). Acid-etched SEM analyses revealed that *Dspp^P19L/+^* mice showed an acid-etching pattern in enamel that was similar to that of the wild-type mice, at the age of 3weeks (Figure 5A). At the age of 24weeks, *Dspp^+/+^* mice presented an acid-etching pattern, with the cross-sectioned enamel rods preferentially removed by acid (Figure 5B). Although *Dspp^P19L/+^* mice displayed a similar pattern, the cross-sectioned enamel rods were not as well-defined as the wild-type mice (Figure 5B). There were no recognizable enamel rods in *Dspp^P19L/P19L^* mice at both ages (Figures 5A,B). In addition, the dentinoenamel junction (DEJ) appeared to be altered in *Dspp^P19L/P19L^* mice, compared to age-matched *Dspp^P19L/+^* and *Dspp^P19L/+^* mice (Figures 5A,B). Taken together, these findings support that the molar enamel was poorly formed in *Dspp^P19L/+^* and *Dspp^P19L/P19L^* mice, particularly in *Dspp^P19L/P19L^* mice.

### An Accumulation of DSPP Protein Within the Presecretory Ameloblasts in *Dspp^P19L/+^* and *Dspp^P19L/P19L^* Mice

**Figure 6:**
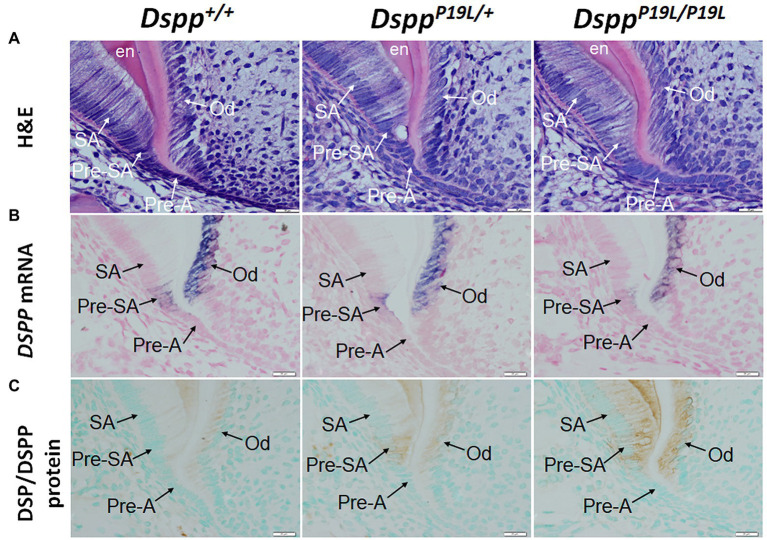
Intracellular accumulation of dentin sialophosphoprotein (DSPP) protein in presecretory ameloblasts. All images are the sagittal section and distal cervical region of the mandibular first molars of 1-week-old wild-type *Dspp^+/+^*, heterozygous *Dspp^P19L/+^* and homozygous *Dspp^P19L/P19L^* mice. **(A)** H&E staining. **(B)** *In situ* hybridization analysis of *DSPP* mRNA (signal in purple). *Dspp^P19L/+^* mice had a moderate decrease, whereas homozygous *Dspp^P19L/P19L^* mice showed a marked decrease in *DSPP* mRNA level in both odontoblasts and presecretory ameloblasts, compared to the wild-type mice. **(C)** Immunohistochemical staining of DSP/DSPP protein (signal in brown). The immunohistochemical staining signals for DSP/DSPP protein were weakly detected in the presecretory ameloblasts and odontoblasts in the wild-type mice. There was a moderate increase in the DSP/DSPP signals in both presecretory ameloblasts and odontoblasts in *Dspp^P19L/+^* mice, and a strong increase in *Dspp^P19L/P19L^* mice. SA, secretory ameloblasts; Pre-SA, presecretory ameloblast, Pre-A, preameloblasts; OD, odontoblasts; and en, enamel. Scale bars: 20μm in A–I.

**Figure 7:**
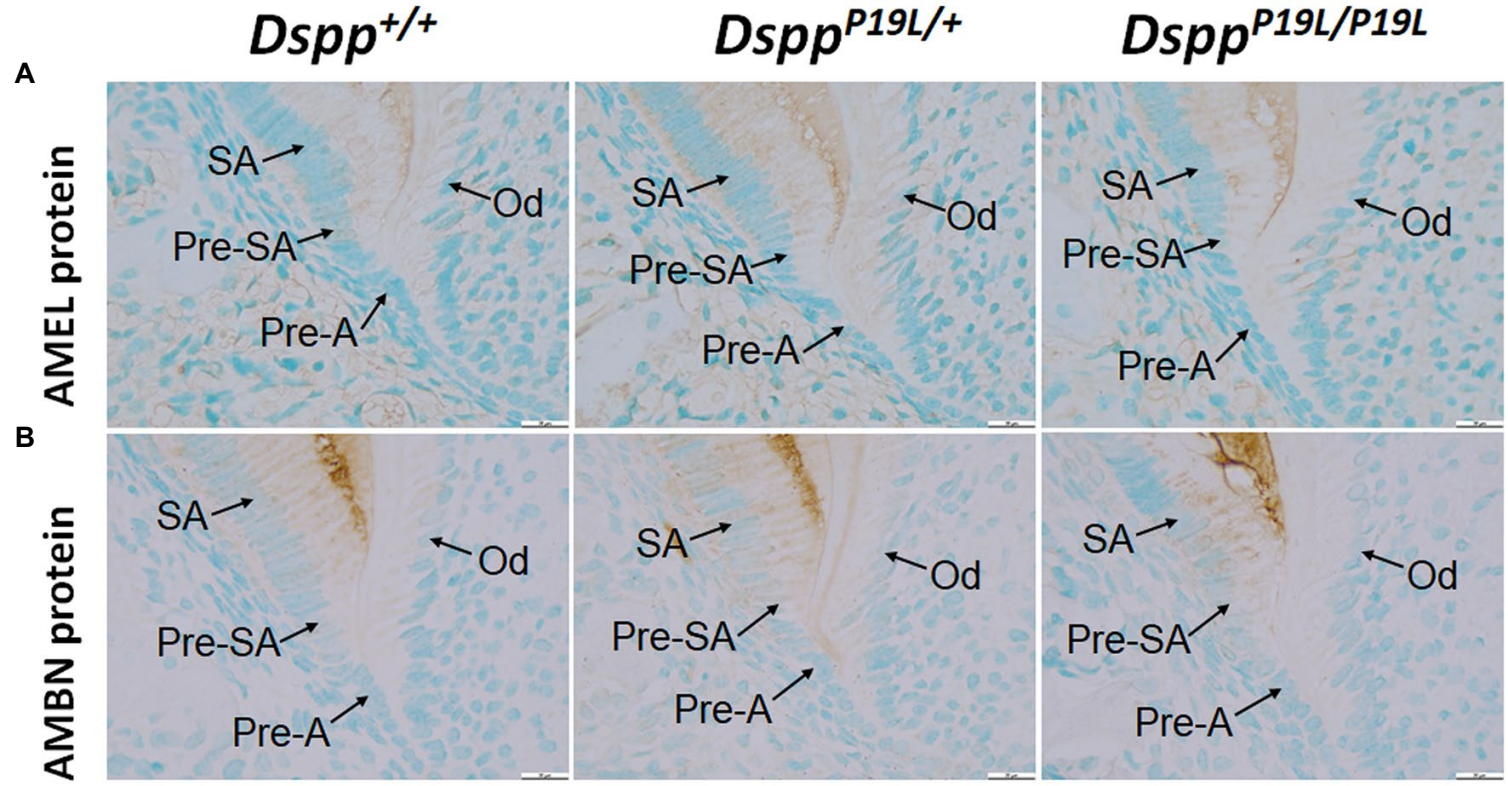
Immunohistochemical staining of amelogenin (AMEL) and ameloblastin (AMBN). All images are the sagittal section and distal cervical region of the mandibular first molars of 1-week-old wild-type *Dspp^+/+^*, heterozygous *Dspp^P19L/+^* and homozygous *Dspp^P19L/P19L^* mice. **(A)** Immunohistochemical staining of AMEL (signal in brown). **(B)** Immunohistochemical staining of AMBN (signal in brown). All three groups of mice showed a similar distribution and intensity of AMEL and AMBN immunostaining signals in the enamel matrices. SA, secretory ameloblasts; Pre-SA, presecretory ameloblast, Pre-A, preameloblasts; and OD, odontoblasts. Scale bars: 20μm in A–F.

To determine the molecular changes that occurred in the differentiating ameloblasts, we examined the expression of the *Dspp* gene at both mRNA and protein levels in the mandibular first molars (pre-eruptive phase) of 7-day-old *Dspp^P19L/+^* and *Dspp^P19L/P19L^* mice. Histologic analyses showed that the secretory ameloblasts in *Dspp^P19L/+^* and *Dspp^P19L/P19L^* mice appeared to be similar in morphology to those in the *Dspp^+/+^* mice (Figure 6A). *In situ* hybridization demonstrated that a low level of *DSPP* mRNAs was observed in the presecretory ameloblasts in *Dspp^+/+^* mice (Figure 6B). Unlike the strong and sustained *Dspp* expression in the opposing odontoblasts, the *Dspp* expression abruptly diminished as the presecretory ameloblasts differentiated into secretory ameloblasts (Figure 6B). Compared to *Dspp^+/+^* mice, there was a moderate decrease in *DSPP* mRNA signal in both presecretory ameloblasts and odontoblasts in *Dspp^P19L/+^* mice and a great decrease in *Dspp^P19L/P19L^* mice (Figure 6B). In contrast to the changes in *DSPP* mRNA, immunohistochemical signals for DSP/DSPP protein were weakly detected in the presecretory ameloblasts and odontoblasts in *Dspp^+/+^* mice (Figure 6C). There was a moderate increase in the DSP/DSPP signals in both presecretory ameloblasts and odontoblasts in *Dspp^P19L/+^* mice, and a strong increase in *Dspp^P19L/P19L^* mice (Figure 6C). Nevertheless, immunohisto chemistry showed that there was no obvious difference in the level and distribution of two secretory stage ameloblast markers, AMEL and AMBN, in the enamel matrices among all three groups of mice (Figures 7A,B). These results suggest that the defective enamel may be associated with an accumulation of the mutant P19L-DSPP protein within the presecretory ameloblasts in *Dspp^P19L/+^* and *Dspp^P19L/P19L^* mice.

## Discussion

The enamel defects are less-well studied in human DGI patients associated with DSPP mutations because of rapid enamel attrition in human patients and lack of an appropriate animal model. We previously generated a mouse model that expresses P19L-DSPP – a mouse equivalent of human mutant P17L-DSPP, and reported the dentin/pulp phenotypes of *Dspp^P19L/+^* and *Dspp^P19L/P19L^* mice. In this study, we presented the ultrastructural enamel defects of these mice as well as the molecular changes in the differentiating ameloblasts and demonstrated that the enamel defects may be associated with an accumulation of the mutant P19L-DSPP protein within the presecretory ameloblasts.

The teeth often undergo rapid and severe attrition in DGI patients associated with DSPP mutations. It is generally believed that enamel readily chips off from the underlying defective dentin and/or abnormal DEJ after tooth eruption, thereby exposing the softer and malformed dentin to rapid attrition (Wright and Gantt, 1985; Kim et al., 2004; MacDougall et al., 2006; Hart and Hart, 2007; Kida et al., 2009; Min et al., 2014; Porntaveetus et al., 2018). However, it has also become evident that in some cases, the enamel itself may have intrinsic developmental defects (Lee et al., 2011; Wang et al., 2011; Bloch-Zupan et al., 2016; Taleb et al., 2018), which may be a direct contributing factor to rapid tooth wear observed in DGI patients. Consistently, we previously reported that both *Dspp^P19L/+^* and *Dspp^P19L/P19L^* mice showed significantly reduced thickness of dental pulp chamber roof dentin and reduced dentin mineral densities (Liang et al., 2019), suggesting that the defective dentin may contribute to the accelerated tooth attrition as it cannot provide adequate support to the overlying enamel. In this study, we further demonstrated that *Dspp^P19L/+^* and *Dspp^P19L/P19L^* mice displayed delayed enamel maturation and reduced enamel mineral densities, corroborating the intrinsic defects in the enamel. Similar to human DGI patients, the malformed enamel experienced accelerated attrition after the teeth erupted in *Dspp^P19L/+^* and *Dspp^P19L/P19L^* mice. These human and animal genetic studies strongly demonstrate that DSPP mutations can cause both enamel and dentin defects, which may together contribute to the severe tooth attrition seen in DGI patients. However, it is of note that some DSPP mutations may have no or negligible effects on enamel formation (McKnight et al., 2008; Nieminen et al., 2011; Verdelis et al., 2016; Yang et al., 2016; Park et al., 2020).

During tooth development, *Dspp* is transiently expressed by the presecretory ameloblasts (D'Souza et al., 1997; Ritchie et al., 1997; Begue-Kirn et al., 1998; MacDougall et al., 1998; Bleicher et al., 1999). Thereby, it has been speculated that the enamel defects may be due to ameloblast pathology caused by mutant DSPP proteins (Lee et al., 2011; Wang et al., 2011). Indeed, we found that even though the level of DSPP mRNA was markedly reduced in the presecretory ameloblasts, and the mutant P19L-DSPP protein was accumulated within the cells in both *Dspp^P19L/+^* and *Dspp^P19L/P19L^* mice. We have previously reported that the mutant P19L-DSPP protein was accumulated in the endoplasmic reticulum when it was transiently expressed in *in vitro* cultured odontoblast-like cells (Liang et al., 2019). Therefore, it is most likely that the mutant P19L-DSPP protein was also retained in the ER within the presecretory ameloblasts. DSPP is highly acidic as it contains a large number of aspartate and glutamate residues (Prasad et al., 2010). If the full-length DSPP was accumulated in the ER, the highly acidic DSPP could incur pathogenic ER stress. It has been shown that ER stress and its associated unfolded protein response (UPR) is involved in AI caused by mutations in the genes that encode enamel matrix proteins (Brookes et al., 2014, 2017a,b; Hetz et al., 2020). Therefore, it is reasonable to postulate that the enamel defects observed in *Dspp^P19L/+^* and *Dspp^P19L/P19L^* mice may be associated with ER stress and its activated UPR caused by mutant P19L-DSPP protein.

It is important to note that even though the mutant *Dspp^P19L^* allele, like the wild-type *Dspp* allele, was only transiently expressed in the presecretory ameloblasts, it caused an irreversible damage on ameloblast differentiation and function. First, we demonstrated that the function of the secretory-stage ameloblasts was affected as evidenced by reduced enamel formation, even though the levels of AMEL and AMBN in the enamel matrices appeared to be normal in *Dspp^P19L/+^* and *Dspp^P19L/P19L^* mice. Secondly, the function of the maturation-stage ameloblasts was also compromised as reflected by delayed enamel maturation in *Dspp^P19L/+^* and *Dspp^P19L/P19L^* mice. These findings further emphasize the notion that the presecretory ameloblasts are very sensitive to their intracellular and extracellular disturbances so that any pathological conditions may affect their function and continual differentiation and cause developmental enamel defects (Lee et al., 2011; Wang et al., 2011). Further studies are needed to determine how the mutant P19L-DSPP protein exerted its negative effect on the presecretory ameloblasts, and their subsequent differentiation into secretory stage ameloblasts and maturation stage ameloblasts.

*Dspp^P19L/+^* and *Dspp^P19L/P19L^* mice manifested an enamel phenotype that is quite different from *Dspp*-null mice. It has been reported that even though *Dspp*-null mice had earlier onset of enamel matrix deposition in mandibular incisors, they did not exhibit any major phenotypic abnormalities in mature enamel with regards to enamel structural organization, mineral density, or hardness (Verdelis et al., 2016). Nevertheless, *Dspp*-null mice did show a defect in DEJ, which might be due to either the loss of DSPP function in the formation of this dentin and enamel interface or the earlier onset of enamel deposition (Verdelis et al., 2016). In contrast, *Dspp^P19L/+^* and *Dspp^P19L/P19L^* mice not only displayed an abnormal DEJ as evidenced by acid-etched SEM analysis, they also had reduced enamel formation as well as intrinsic structural defects in enamel. Moreover, unlike *Dspp* heterozygous mice who did not show any apparent dental phonotype (Sreenath et al., 2003; Suzuki et al., 2009; Verdelis et al., 2016), our current and previous studies showed that the *Dspp^P19L/+^* mice developed a similar, though less severe, tooth phenotype as *Dspp^P19L/P19L^* mice (Liang et al., 2019). The phenotypic difference between the *Dspp* mutant mice and *Dspp*-null mice support that the enamel phenotypes of *Dspp^P19L/+^* and *Dspp^P19L/P19L^* mice were not simply caused by a loss of DSPP function in the presecretory ameloblasts.

Moreover, previous studies have shown that ectopic overexpression of DSP in secretory ameloblasts in mice resulted in an increase in enamel hardness, whereas similar overexpression of DPP weakened enamel and made it more prone to wear (Paine et al., 2005; White et al., 2007). Yet, it is very unlikely that the enamel phenotypes of *Dspp^P19L/+^* and *Dspp^P19L/P19L^* mice were caused by ectopic overexpression of DSP or DPP. First, we have demonstrated that the mutant *Dspp^P19L^* allele, like the wild-type *Dspp* allele, was only transiently expressed in the presecretory ameloblasts. Secondly, our previous studies have demonstrated that although the secretion of P19L-DSPP was impaired, Western-blotting analysis of the total proteins extracted from the dental pulps and dentin matrices of first molars of *Dspp^P19L/P19L^* mice as well as the proteins from the conditioned media harvested from cells transfected with the mutant P19L-DSPP-expressing construct revealed that the mutant P19L-DSP/DSPP proteins showed a similar migrating pattern as the normal DSP/DSPP proteins, suggesting that the secreted mutant and normal DSPP proteins were subject to similar posttranslational modifications and proteolytic processing (Liang et al., 2019). Therefore, it is reasonable to exclude the possibility that the enamel phenotypes of the *Dspp* mutant mice were caused by a gain of DSP or DPP function in the presecretory/secretory ameloblasts.

In summary, we have demonstrated that DSPP mutations could affect enamel formation and cause severe intrinsic enamel defects. Future studies are warranted to determine the molecular pathogenesis underlying the enamel defects associated with the mutant P19L-DSPP protein.

## Data Availability Statement

The original contributions presented in this study are included in the article/supplementary material; further inquiries can be directed to the corresponding author.

## Ethics Statement

The animal study was reviewed and approved by the Institutional Animal Care and Use Committee of Texas A&M University College of Dentistry.

## Author Contributions

TL and QX contributed to design, data acquisition, analysis and interpretation, drafted, and critically revised the manuscript. HZ and SW contributed to data acquisition, analysis, interpretation, drafted, and critically revised the manuscript. TD and CQ contributed to interpretation, drafted, and critically revised the manuscript. YL contributed to conception, design, interpretation, drafted, and critically revised the manuscript. All authors approved the final version of the submitted manuscript and agree to be accountable for all aspects of the work.

## Funding

This work was supported by National Institute of Dental & Craniofacial Research (NIDCR) grant DE027345.

## Conflict of Interest

The authors declare that the research was conducted in the absence of any commercial or financial relationships that could be construed as a potential conflict of interest.

## Publisher’s Note

All claims expressed in this article are solely those of the authors and do not necessarily represent those of their affiliated organizations, or those of the publisher, the editors and the reviewers. Any product that may be evaluated in this article, or claim that may be made by its manufacturer, is not guaranteed or endorsed by the publisher.
